# Characterization of Undiscovered miRNA Involved in Tumor Necrosis Factor Alpha-Induced Atrophy in Mouse Skeletal Muscle Cell Line

**DOI:** 10.3390/ijms25116064

**Published:** 2024-05-31

**Authors:** Dominika Pigoń-Zając, Marcin Mazurek, Mirosław Maziarz, Michael Ochieng’ Otieno, Javier Martinez-Useros, Teresa Małecka-Massalska, Tomasz Powrózek

**Affiliations:** 1 Department of Human Physiology of the Chair of Preclinical Sciences, Medical University in Lublin, 20-080 Lublin, Poland; pigon.dominika@gmail.com (D.P.-Z.); marcinmazurek1212@gmail.com (M.M.); miroslawjanmaziarz@gmail.com (M.M.); tmalecka@gmail.com (T.M.-M.); 2 Translational Oncology Division, Oncohealth Institute, Fundacion Jiménez Díaz University Hospital, 28040 Madrid, Spain; mikeotson@yahoo.com (M.O.O.); javier.museros@quironsalud.es (J.M.-U.); 3 Area of Physiology, Department of Basic Health Sciences, Faculty of Health Sciences, Rey Juan Carlos University, 28922 Madrid, Spain

**Keywords:** muscle atrophy, TNF-α, inflammation, miRNA, myotubes

## Abstract

Muscular atrophy is a complex catabolic condition that develops due to several inflammatory-related disorders, resulting in muscle loss. Tumor necrosis factor alpha (TNF-α) is believed to be one of the leading factors that drive inflammatory response and its progression. Until now, the link between inflammation and muscle wasting has been thoroughly investigated, and the non-coding RNA machinery is a potential connection between the candidates. This study aimed to identify specific miRNAs for muscular atrophy induced by TNF-α in the C2C12 murine myotube model. The difference in expression of fourteen known miRNAs and two newly identified miRNAs was recorded by next-generation sequencing between normal muscle cells and treated myotubes. After validation, we confirmed the difference in the expression of one novel murine miRNA (nov-mmu-miRNA-1) under different TNF-α-inducing conditions. Functional bioinformatic analyses of nov-mmu-miRNA-1 revealed the potential association with inflammation and muscle atrophy. Our results suggest that nov-mmu-miRNA-1 may trigger inflammation and muscle wasting by the downregulation of LIN28A/B, an anti-inflammatory factor in the let-7 family. Therefore, TNF-α is involved in muscle atrophy through the modulation of the miRNA cellular machinery. Here, we describe for the first time and propose a mechanism for the newly discovered miRNA, nov-mmu-miRNA-1, which may regulate inflammation and promote muscle atrophy.

## 1. Introduction

Muscle wasting followed by muscular insufficiency are noticeable signs of skeletal muscle atrophy, and they mainly appear from a progressive imbalance between protein synthesis and degradation [1]. Skeletal muscle loss is frequently observed under different pathological conditions related to an abnormal and long-lasting inflammatory response of the body through the activation of receptor-mediated intramuscular signaling pathways, such as nuclear factor kappa-light-chain-enhancer of activated B cells (NF-κB) [2]. In a skeletal muscle, this signaling pathway is directly promoted by tumor necrosis factor alpha (TNF-α) under inflammatory conditions. It is also activated by various inflammatory markers synthesis, including pro-inflammatory cytokines: interleukin-1 and -6 (IL-1, IL-6), which subsequently activate ubiquitin-dependent proteolysis and myotube apoptosis [3,4]. Moreover, NF-κB signaling significantly inhibits the expression of muscle differentiation-related genes encoding mainly myoblast determination protein 1 (MyoD) and myogenin (MYOG), making muscle regeneration impracticable [5,6].

The exact process of muscle atrophy is still not fully understood due to the significant number of cellular pathways involved and the complexity of interaction between factors mediating muscle loss [7]. A close relationship between inflammation and muscular atrophy turned attention to investigating factors that could be involved in mediation and interaction between both conditions [8]. Among them, some molecular markers, such as miRNAs, which belong to the non-coding RNAs family, are attractive candidates to link inflammatory response with muscle atrophy [9]. It is believed that miRNAs are crucial for controlling skeletal muscle development and metabolism. Thus, their alteration can cause muscular imbalance. For instance, miRNA-1, 133, 206, and 29b can influence myocyte growth and wasting [10]. Due to their ability to regulate skeletal myogenesis and muscle regeneration processes, miRNA-1 and miRNA-133 family members significantly influence skeletal muscle physiology. According to recent in vitro research, the TNF-α inhibitory impact on muscle function can be mediated by a few regulatory miRNAs, such as miRNA-155 and miRNA-503 [11,12]. It was reported that miRNA-155 was downregulated and miRNA-503 was upregulated in normal muscle promoting proper myotube formation and myoblast development. However, inflammation followed by increased TNF-α inversely regulates their expression, consequently inhibiting muscle differentiation [12,13,14].

The influence of TNF-α, a leading pro-inflammatory factor on muscle function mediated by miRNAs, is still under study. Nevertheless, the discovery of novel miRNAs could broaden the knowledge of the inflammatory-dependent muscle atrophy process and identify its putative biomarkers. This study aimed to identify novel miRNAs potentially involved in inflammatory-related muscle wasting by the TNF-α-induced atrophy model of the murine C2C12 cell line.

## 2. Results

### 2.1. Atrophy Model Increased FBXO32 and MuRF1 Levels In Vitro

**Figure 1:**
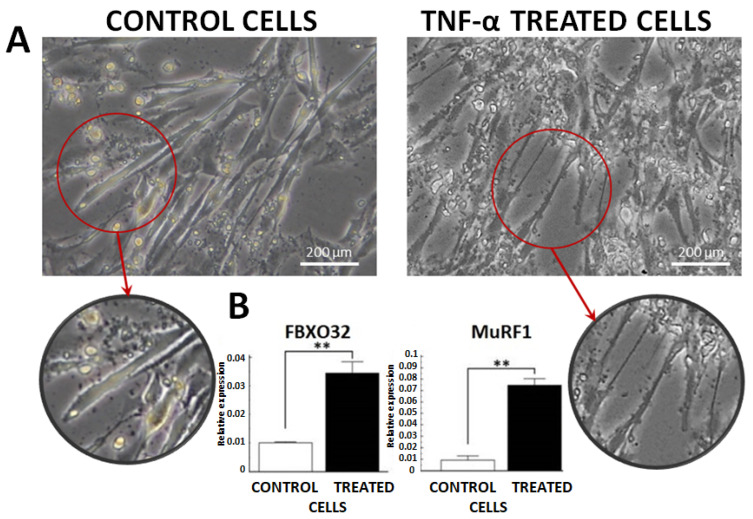
Model of C2C12 myotube atrophy. (**A**) Observable differences in myotube morphology under a microscope examination between untreated cells (well-differentiated myotubes) and C2C12 cell culture treated with 100 ng/mL TNF-α within 72 h (atrophy model). (**B**) Differences in the atrophy marker expressions—*FBXO32* and *MuRF1*—between the control and TNF-α treated cells. (**—*p* < 0.001).

There were differences in C2C12 cell morphology under microscope examination, including well-differentiated myotubes (control cells) and atrophy-induced models 72 h after treatment with 100 ng/mL of TNF-α (treated cells) (Figure 1A). We noticed highly significant differences in the expression of atrophy markers F-Box Protein 31 and Muscle Ring-Finger Protein-1 gene (*FBXO32* and *MuRF1*) between the control and treated cells—about a three-fold and seven-fold increase, respectively (Figure 1B).

### 2.2. DEnov-miRNA and DEmiRNA Profiling Highlights Undiscovered miRNAs Associated with Muscle Atrophy

**Figure 2:**
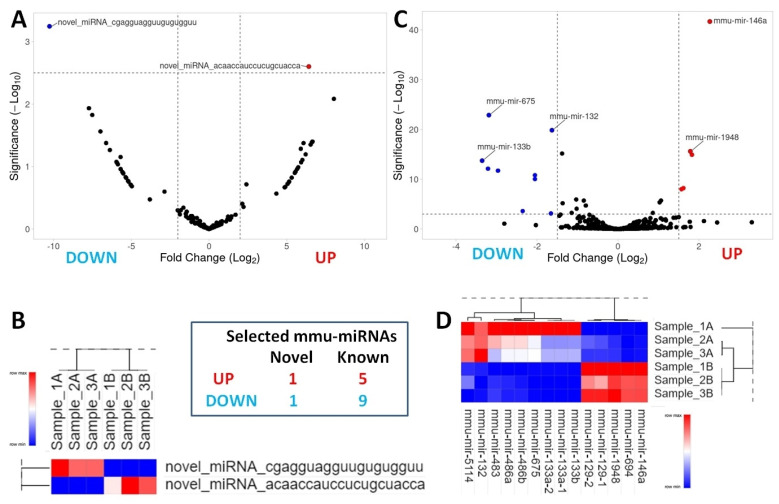
Identification of DEnov-miRNAs and DEmiRNAs between treated and control C2C12 cultures by next-generation sequencing (NGS) in a discovery stage. (**A**) Volcano plot illustrating differences in expression of 175 novel mmu-miRNAs between studied cultures—the significant difference in the expression of the two molecules was recorded for the samples. (**B**) Heat map of the two DEnov-miRNAs, demonstrating the considerable difference in expression among the tested samples. (**C**) Volcano plot illustrating the differences in the expression of 868 known mmu-miRNA sequences between the studied cultures—the significant difference is the expression of the top 14 molecules. (**D**) Heat map illustrating expression differences of the top 14 DEmiRNAs between treated C2C12 myotube cultures and control cells. Above all, 16 mmu-miRNAs were selected in the discovery stage: 6 miRNAs were upregulated and 10 downregulated.

There were 175 new differentially expressed mmu-miRNAs (DEnov-miRNAs; 86 downregulated and 89 upregulated) identified in treated cultures, and they demonstrated altered expression compared with untreated C2C12 cells. The most significant DEnov-miRNAs with large fold changes and extreme statistical significance were identified and visualized by a volcano plot (Figure 2A). The following two nov-mmu-miRNAs were revealed as prospective, and the most significant molecules for further analysis were nov-mmu-miRNA-1: 5′-cgagguagguugugugguu-3′ (downregulated) and nov-mmu-miRNA-2: 5′-acaaccauccucugcuacca-3′ (upregulated). A generated heat map illustrated differences in the expression of nov-miRNAs between the treated and untreated cells with a hierarchical clustering–tested discovery sample set that included analysis of three untreated cultures (samples 1A–3A) and three cell lines treated with TNF-α (samples 1B–3B) (Figure 2B). Additionally, we found 475 downregulated and 393 upregulated known miRNA sequences (868 DEmiRNAs) in treated cultures in contrast to untreated ones. Volcano plot analysis revealed 75 miRNAs with high significance. However, the top 14 miRNAs were eventually selected as the most significant molecules varying in expression between samples. Among them, nine miRNAs were downregulated (mmu-miRNA: 675, 132, 133a-1, 133a-2, 133b, 486a, 486b, 483, and 5114) and five were upregulated (mmu-miRNA: 146a, 1948, 694, 129-1, and 129-2) (Figure 2C). A heat map illustrating differences in the expression of known miRNAs between the treated and untreated cells is shown in Figure 2D.

### 2.3. DEnov-miRNAs Were Validated under the Different Treatment Conditions

**Figure 3:**
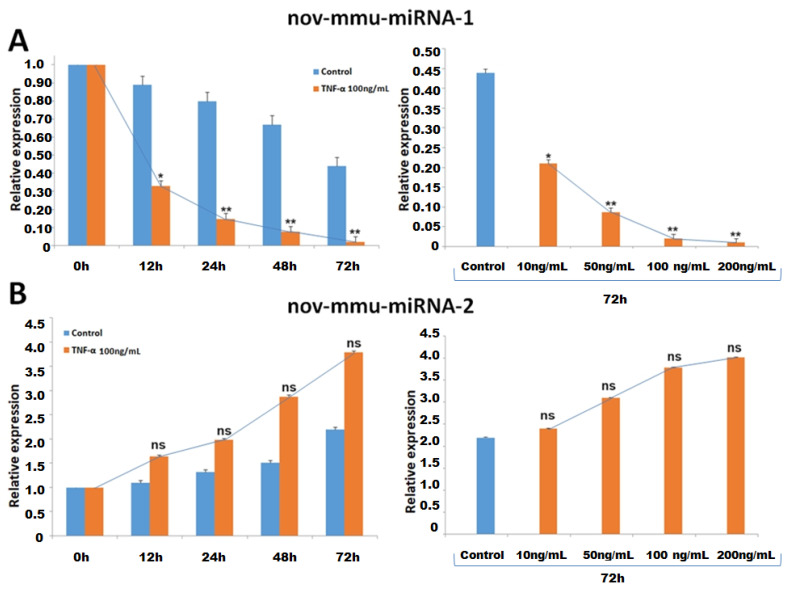
Result of validation of the identified DEnov-miRNAs by qRT-PCR. (**A**) Differences in nov-mmu-miRNA-1 expression between the control and TNF-α-treated C2C12 cells at different time intervals (12, 24, 28, and 72 h, respectively) and differences in miRNA expression after 72 h of cell growth between the control and treated cells treated with different TNF-α concentrations (10, 50, 100, and 200 ng/mL, respectively). (**B**) Differences in nov-mmu-miRNA-2 expression between the control and TNF-α-treated C2C12 cells at different time intervals (12, 24, 28, and 72 h, respectively) and differences in the miRNA expression after 72 h of cell growth between the control and treated cells treated with different TNF-α concentrations (10, 50, 100, and 200 ng/mL, respectively) (* *p* < 0.05; ** *p* < 0.001; ns—non-significant).

The two DEnov-miRNA identified in the discovery stage were independently validated by qRT-PCR in a new set of control C2C12 cultures and cells treated with TNF-α at different time intervals and various cytokine concentrations (Figure 3). This approach allowed us to monitor the dynamic changes of expressions of both studied miRNAs in normal myotubes and atrophy models. We noticed a gradual decrease in nov-mmu-miRNA-1 expression in the control and cells treated with 100 ng/mL of TNF-α at different time intervals. Interestingly, the dynamic change in the miRNA expression of TNF-α-treated cells was significant from the first condition evaluated. The greatest change in its expression was recorded between 48 h and 72 h after the cell culture (3.7-fold change; *p* < 0.001). The miRNA expression shown as a fold change in the treated cells was 2.69-fold (*p* < 0.05), 5.37-fold, 8.70-fold, and 20.9-fold (*p* < 0.001) lower compared to untreated cells after 12 h, 24 h, 48 h, and 72 h of culturing, respectively. Also, the various TNF-α treatment concentrations influenced the nov-mmu-miRNA-1 expression level 72 h after culturing. An inverse moderate correlation between cytokine concentration and the expression of the miRNA under study was noticed. On the one hand, following the miRNA expression throughout various TNF-α concentrations, the most significant decrease in its expression was observed in the case of increased cytokine concentration from 50 ng/mL up to 100 ng/mL (4.2-fold decrease in miRNA expression). Comparing the expression of nov-mmu-miRNA-1 between cells treated with different TNF-α concentrations and control cells measured after 72 h of culturing, the following fold changes were recorded: 10 ng/mL (2.1-fold; *p* < 0.05), 50 ng/mL (5.0-fold; *p* < 0.001), 100 ng/mL (20.9-fold; *p* < 0.001), and 200 ng/mL (38.9-fold; *p* < 0.001) (Figure 3A). The discussed phenomena have not been recorded for nov-mmu-miRNA-2 (Figure 3B).

### 2.4. Characterization of Nov-mmu-miRNA-1 Revealed Its Involvement in Several Biological Processes

**Figure 4:**
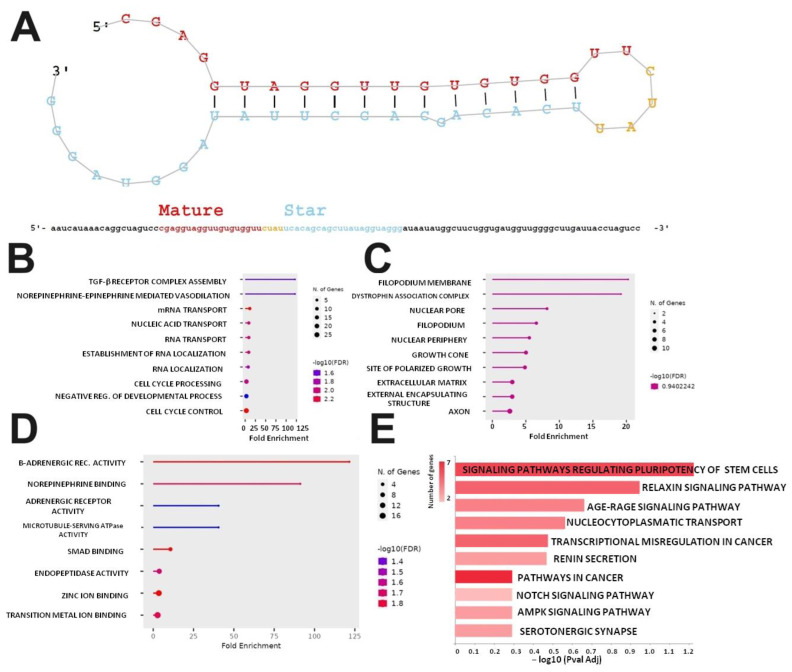
Structural and functional analysis of nov-mmu-miRNA-1. (**A**) RNA secondary structure of nov-mmu-miRNA-1 (the mature sequence is highlighted in red, and the star sequence is in blue) and the result of GO enrichment analysis—the top 10 terms were presented for the biological process. (**B**) Cellular component. (**C**) Molecular function. (**D**) The result of KEGG enrichment analysis. (**E**) The top 10 enriched terms.

Nov-mmu-miRNA-1 is a miRNA located on chromosome 3q, and the mature sequence consists of 19 nucleotides in length. The secondary structure of its precursor is presented in Figure 4A. Sequence analysis of the studied molecule conducted by the RNA central tool revealed its distant similarity to the mmu-let-7 miRNA family, mainly let-7a, b, 7c, 7c-1, 7c-2, and 7e (it is most likely to the mmu-let-7b-5p sequence with about 68% sequence similarity). However, its sequence has not been described so far. Using the miRDB tool, the potential targets for nov-mmu-miRNA-1 were predicted. In all, 806 potential gene targets were found. However, 120 (14.9%) demonstrated a prediction score of at least 90%. Additionally, 661 gene targets were identified for let-7b, and 149 (26.6%) of them demonstrated a prediction score of >90%. We identified 285 common gene targets for both miRNAs; however, 376/661 genes (56.9%) were exclusive for let-7b, whereas 521/806 (64.6%) were for nov-mu-miRNA-1. The 120 most likely targeted by nov-mmu-miRNA-1 genes were introduced to GO and KEGG analysis to define the potential biological function of this novel miRNA. According to the GO enrichment analysis, the cell cycle (FDR = 4.43 × 10^−3^), mRNA transport (FDR = 4.40 × 10^−3^), and nucleic acid transport (FDR = 9.50 × 10^−3^) were the most enriched GO terms related to the biological processes (BP). The nucleus (FDR = 4.57 × 10^−3^) for cellular component (CC) and beta-adrenergic receptor activity (FDR = 1.30 × 10^−2^), zinc ion binding (FDR = 1.30 × 10^−2^), and SMAD binding (FDR = 1.32 × 10^−2^) were the most enriched GO terms related to for the molecular function (MF). The top, most significantly enriched GO terms, including BP, MF, and CC, are presented in Figure 4B–D. According to the KEGG enrichment analysis, the two most significantly enriched pathways were pathways regulating the pluripotency of stem cells (FDR = 4.26 × 10^−2^) and relaxing in signaling pathways (FDR = 0.011) (Figure 4E). Interestingly, WikiPathways analysis postulated the involvement of the studied mmu-miRNA into pathways, regulating inflammatory response by the regulation of particular genes, such as the MAPK signaling pathway (5/158 genes), the inflammatory response pathway (1/30 genes), cytokines and inflammatory response (1/27 genes), and the TNF-α and NF-κB signaling pathway (5/183 genes). Moreover, the MGI tool uncovered a linkage between genes targeted by nov-mmu-miRNA-1 and the following terms related to altered muscle function: abnormal muscle morphology (7/71 genes), abnormal skeletal muscle fiber size (2/5 genes), muscle fatigue (2/12), and abnormal skeletal muscle satellite cell proliferation (3/12 genes).

**Figure 5:**
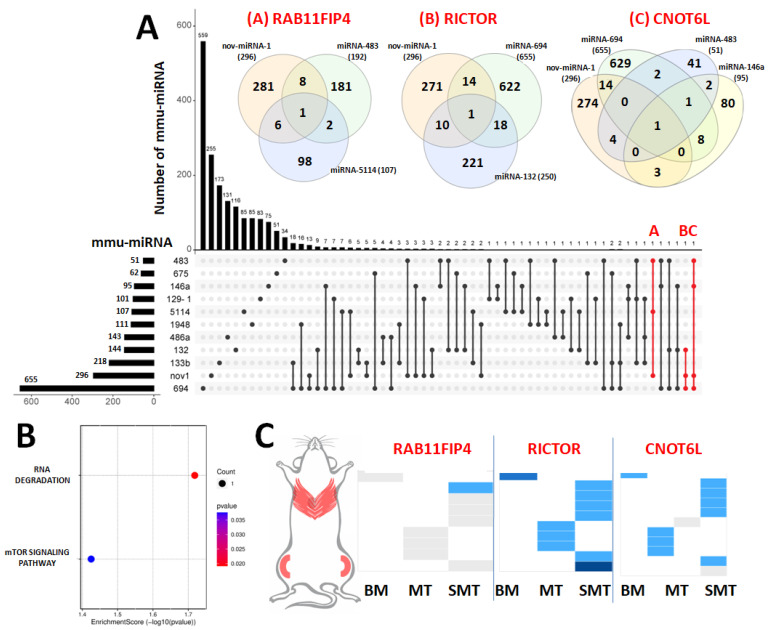
UpSet plot highlights several mutual targets for identified DEmiRNAs. (**A**) Horizontal bars represent several gene targets for the individual miRNAs, and the vertical bars demonstrate individual or mutual gene targets for intersection. Points and connecting lines in the matrix indicate sets that are part of the intersection. Red dots and the connecting lines demonstrate mutual gene targets between nov-mmu-miRNA-1 and at least two other selected DEmiRNAs. The three sets meeting these criteria were identified and summarized in the Venn diagrams (joint targets plot A—RAB11FIB4, plot B—RICTOR, and plot C—CNOT6L). (**B**) KEGG pathway enrichment analysis for the three genes targeted by nov-mmu-miRNA-1 and at least two other miRNAs. (**C**) Varied mice muscle tissue expression of *RAB11FIP4*, *RICTOR*, and *CNOT6L*—the columns in the chart represent gene expression in BM—biceps muscle, MT—muscle tissue, and SMT—skeletal muscle tissue. In contrast, the rows demonstrate their expression in the different study sets. The colors of cells refer to expression (white—no evidence, gray—very low, light blue—low, and deep blue—moderate).

Bioinformatics analysis was used to investigate the mutual gene targets between the selected known DEmiRNAs (presented in Figure 2D) and nov-mmu-miRNA-1. An UpSet plot was generated to demonstrate the intersections between miRNA targets (restricted to targets representing at least 80% binding score). As presented in Figure 5A, specific miRNAs can target a great number of genes, which are not shared with other investigated miRNAs and are illustrated as single dots in the UpSet plot (no intersections). We found thirty-nine intersections created between two different miRNAs (the number of the mutual targets ranged between one and eighteen) and ten intersections created between three different miRNAs; however, the number of commonly targeted genes for these intersections was small (only one or two gene targets) and are illustrated as dots and connecting lines in the plot. The main goal of this analysis was to reveal as large as possible intersections between nov-mmu-miRNA-1 and other selected miRNAs, and, afterwards, find mutual gene targets for them. This approach could identify a group of cooperating miRNAs participating in the joint regulation of genes involved in muscular function under the inflammatory condition. As mentioned above, the greatest identified intersections contain common gene target maximums for three miRNAs, and we revealed only three of such intersection sets that covered nov-mmu-miRNA-1 (dots and connecting red lines, as well as Venn diagrams (Figure 5A)). The three following intersections represent mutual gene targets for miRNAs. First the intersection between nov-mmu-miRNA-1, mmu-miRNA-5114, and mmu-miRNA-483 (common gene target: *RAB11FIP4*) (Figure 5A; plot A). Second, the intersection between nov-mmu-miRNA-1, mmu-miRNA-694, and mmu-miRNA-133b (common gene target: *RICTOR*) (Figure 5A; plot B). Third, the intersection between nov-mmu-miRNA-1, mmu-miRNA-694, mmu-miRNA-146a, and mmu-miRNA-483 (common gene target: *CNOT6L*) (Figure 5A; plot C). Introducing the three identified common gene targets (*RAB11FIP4*, *RICTOR*, and *CNOT6L*) into KEGG pathway analysis allowed for the prediction of putative pathways related to muscular function in which nov-mmmu-miRNA-1 participates along with other miRNAs. KEGG revealed that the RNA degradation and the mTOR signaling pathway were significantly enriched terms (*p* < 0.05) related to muscular function (Figure 5B). The selected genes, according to the data available in the Expression Atlas [15], demonstrate varied expression levels across the mice skeletal muscle tissues—from very low (*RAB11FIP4*), low (*CNOT6L*), and moderate expression levels (*RICTOR*) (Figure 5C).

### 2.5. Nov-mmu-miRNA-1 Is Associated with Muscle Wasting through a Pro-Inflammatory Action

Based both on the data provided by bioinformatics for identified nov-mmu-miRNA-1 and further screening of the available literature data regarding the regulation of inflammatory conditions, we postulated the putative pro-inflammatory action of nov-mmu-miRNA-1. Under inflammation and the overproduction of pro-inflammatory cytokine, the TNF-α binds to the surface TNFR1 receptor on muscle cells and then initiates cell death events and inflammatory processes by classical NF-κB, MLKL, and Caspase-8 pathways. Prolonged inflammatory conditions initiated by TNF-α in muscle cells cause apoptosis, tissue degradation, proteolysis, inhibited cell regeneration, and prospective atrophic conditions. We assume the indirect impact of TNF-α on nov-mmu-miRNA-1 expression because we did not find evidence in bioinformatics indicating a direct linkage between miRNA and this cytokine. In the events caused by the TNF-α overproduction of IL-6, LIN28A and B are also noticed, and all of the mentioned proteins are encoded by genes targeted by nov-mmu-miRNA-1. During myocyte inflammation, nov-mmu-miRNA-1 is probably not able to keep control over the expression of multiple genes simultaneously; thus, a gradual increase in the expression of numerous genes under the TNF-α can cause the depletion of the studied miRNA and its protective feature of myocyte function. Increased utilization of miRNAs caused by binding with multiple gene targets allows inflammatory conditions to get out of control.

**Figure 6:**
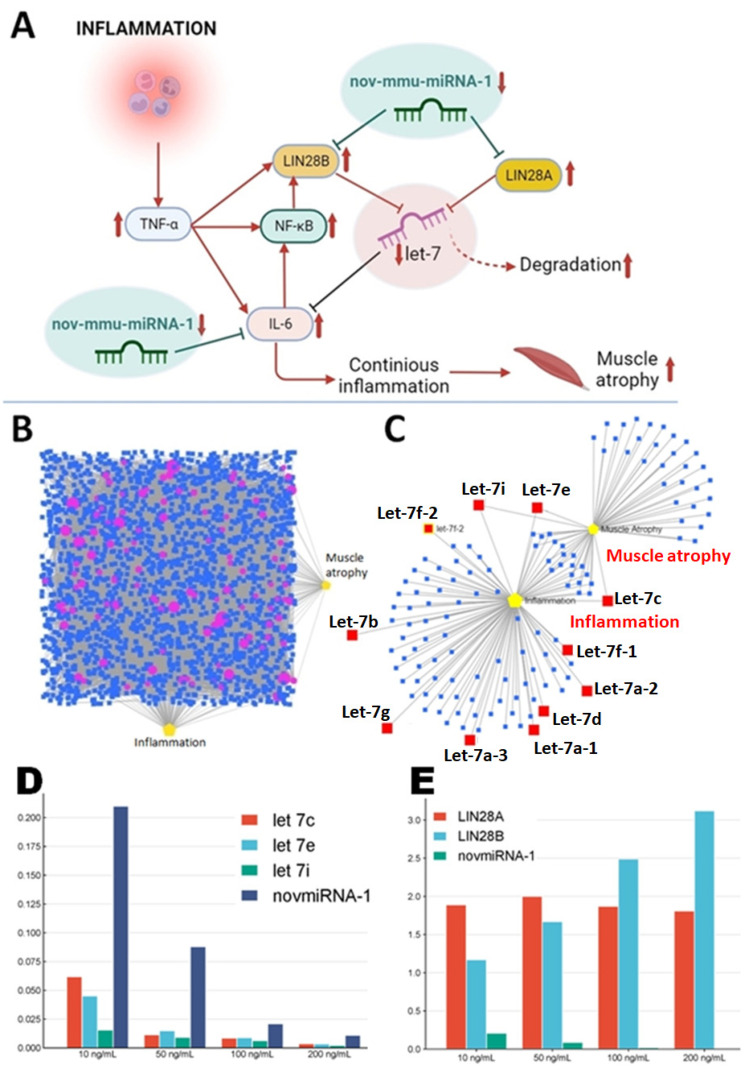
Potential link between nov-mmu-miRNA-1, muscle wasting, and its pro-inflammatory action. (**A**) Proposed mechanism of nov-mmu-miRNA-1 action leading to continuous inflammatory response and muscle wasting. (**B**) Molecular network of interactions between genes targeted by nov-mmu-miRNA-1 (purple dots), other miRNAs targeting those genes (blue squares), and inflammatory and atrophic conditions (yellow marks). (**C**) miRNAs extracted from the molecular network demonstrating selective involvement in the modulation of inflammatory and atrophic conditions (red squares represent let-7 family members indirectly regulated by nov-mmu-miRNA-1. (**D**) Significant expression changes of let-7c,e,i and nov-mmu-miRNA-1 under the different TNF-α concentrations. (**E**) Changes in LIN-28A and LIN-28B expression under the different TNF-α concentrations.

During inflammation, nov-mmu-miRNA-1 wastes its anti-inflammatory action along with let-7 family members, enhancing the synthesis of pro-inflammatory cytokines, mainly IL-6 (Figure 6A). The let-7 family miRNAs appear critical for the progression of inflammation. However, its members can have significant contradictory impacts on such processes. For instance, let-7 inhibits the NF-κB pathway under physiological conditions throughout IL-6 modulation and Toll-like receptor (TLR) pathways. Its biosynthesis and maturation are precisely controlled by LIN28A and LIN28B proteins [16,17,18,19]. Target prediction analysis revealed genes encoding the above-mentioned proteins as highly conserved sites for nov-mmu-miRNA-1 binding (98% and 99% prediction scores, respectively). Under the inflammatory conditions triggered by increased TNF-α concentration, we noticed a decreased expression of the studied miRNA. As presented in Figure 6A, the downregulation of nov-mmu-miRNA-1 can be an additional factor leading to the upregulation of LIN28A and LIN28B, which results in decreased synthesis of let-7, its progressive destruction, and increased IL-6 production. Interestingly, the studied miRNA also targets the gene of IL-6 (target prediction score of 88%), and perhaps the downregulation of nov-mmu-miRNA-1 can be an additional phenomenon causing IL-6 overproduction and continuous inflammatory response. Therefore, the loss of nov-mmu-miRNA-1 function can result in the silencing of let-7, continuous NF-κB pathway activation, and pro-inflammatory cytokine production, which are involved in the development of atrophy in muscle tissue.

Additionally, using the miRNet 2.0 tool, we created a network of molecular interactions between genes targeted by nov-mmu-miRNA-1 (120 genes demonstrating a target score of at least 90%), other miRNAs targeting those genes, and the following terms: inflammation and muscle atrophy (Figure 6B). The created network revealed a close linkage between genes targeted by nov-mmu-miRNA-1 and inflammatory response and the atrophy process that allows one to consider the studied miRNA as an essential player for these conditions. We then extracted only miRNAs related to inflammation or muscle atrophy from the designed molecular network for both conditions. Interestingly, we found several let-7 family members that are involved in the modulation of both phenomena. For instance, let-7c, 7e, and 7i are mutual for inflammatory response and muscular atrophy. This allowed us to hypothesize the involvement of nov-mmu-miRNA-1 in the modulation of the inflammatory response and muscle atrophy via indirect silencing of the mentioned let-7 family members by increasing LIN28A and LIN28B expression. (Figure 6C).

We evaluated changes in let-7c,e,i and LIN-28A and B expression under the different TNF-α treatment conditions to validate our hypothesis. We did not find a significant difference in their expression after treatment with 100 ng/mL of TNF-α at 12 h, 24 h, 48 h, and 72 h time intervals of culture (*p* > 0.05). However, we noticed that the expression of the let-7 family and LIN-28B is affected by the concentration of TNF-α but not LIN-28A. The significant and gradual decrease in let-7c,e,i and the corresponding decrease in nov-mmu-miRNA-1 expression (*p* < 0.05) was related to increased cytokine concentration. Comparing the fold changes between 10 ng/mL and 200 ng/mL, the following were recorded: 17.2 for let-7c, 13.2 for let-7e, and 7.1 for let-7i (Figure 6D). Regarding the fold change of LIN-28A, it was 0.9 (*p* > 0.05) and 2.7 for LIN-28B (*p* < 0.05) (Figure 6E).

## 3. Discussion

Skeletal muscle atrophy can result from numerous pathological conditions. It is typically associated with increased proteolysis, apoptosis, inflammation, and mitochondrial dysfunction. According to the current hypothesis, inflammation plays a significant role in the onset and progression of skeletal muscle wasting [20]. Recently accumulated evidence indicates that a number of cytokines, most notably the pro-inflammatory cytokine, TNF-α, may promote muscular atrophy [21,22]. Inflammatory response and muscle condition undergo complex regulation by non-coding RNA machinery. Thus, it is believed that they could link both conditions in one interdependent process. Although the available literature reports have revealed several aberrantly expressed miRNAs in the process of skeletal muscle atrophy, the precise mechanisms by which those miRNAs function have not been revealed so far [9,23].

Meyer et al. discovered that during myogenic differentiation, the addition of TNF-α significantly upregulates the expression of some miRNAs, such as mmu-miRNA-146a-5p, 202-3p, and 721, while it downregulates others, such as mmu-miRNA-133b-3p, 137-3p, 409-3p, 434-5p, 503-5p, and 542-3p [12]. Moreover, Fiorillo et al. reported that stimulation myotubes with TNF-α increased miRNA-223 and miRNA-146a expression [24]. In another study, Meltzer et al. examined the expression of miRNA-133 in the C2C12 cell line after TNF-α stimulation compared to untreated cell lines. They observed a significant decrease in the expression of miR-133 and miR-1in myoblasts affected by TNF-α [25]. The inflammatory transcription factor NF-κB probably controls the expression of various miRNAs [26,27]. A study conducted by Pangulari et al. showed that the pro-inflammatory influence of a TNF-like weak inducer of apoptosis (TWEAK) inhibits the expression of muscle-specific miR-1 and miR-133a. TWEAK also significantly increased miR-146a expression in TWEAK-treated C2C12 myotubes [28,29]. Hitachi et al. observed a reduced cross-sectional area of muscle fibers after miRNA-486 knock-down in C2C12 cells [30,31]. The results of our study about DEmiRNAs follow these findings. In our data set, we also mainly noticed the substantial overexpression of miRNA-146a and the downregulation of the miRNA-133 family and miRNA-486a/b that may be associated with the modulation of inflammatory-related wasting of muscles.

We have identified a nov-mmu-miRNA-1 sequence whose cellular function has not been previously described. Its sequence suggests a distant similarity to the let-7 family. However, the similarity score accounts for only 68%. This middling number suggests that nov-mmu-miRNA-1 is a novel and unique molecule whose expression depends on TNF-α concentration and curation. Bioinformatics uncovered a potential linkage between genes targeted by nov-mmu-miRNA-1 and the following terms related to altered muscle function: abnormal muscle morphology, abnormal skeletal muscle fiber size, muscle fatigue, and abnormal skeletal muscle satellite cell proliferation. Pathway analysis suggests the involvement of nov-mmu-miRNA-1 in the MAPK signaling pathway, inflammatory response, cytokines and inflammatory response, and the TNF-α-NF-κB signaling pathway. Identified nov-miRNA can either cooperate or compete for gene targets. We identified the following genes regulated by nov-miRNA-1 and at least two additional DEmiRNAs: *RAB11FIP4*, *RICTOR*, and *CNOT6L*. Those genes are expressed across mice’s muscles and are involved in muscular function via the RNA degradation process and mTOR signaling, a key regulator in maintaining skeletal muscle mass [32]. Interestingly, a low expression of nov-miRNA-1, miRNA-483, and miRNA-5114 can facilitate the overexpression of *RAB11FIP4*, whose expression level is low in healthy mice muscle tissues. According to the available literature data, the overexpression of *RAB11FIP4* influences the upregulation of inflammatory cytokines and provokes the activation of the FAK/Akt/NF-κB pathway [33,34,35]. Abnormal *RAB11FIP4* expression was also found in diverse cancer types, such as esophageal cancer, skin cancer, breast cancer, and colorectal cancer. The overexpression of *RAB11FIP4* is associated with multiple biological processes of malignant tumors, including tumor formation, progression, and poor prognosis [34].

As previously described, nov-mmu-miRNA-1 may be an important regulator of inflammation, leading to muscle atrophy. Regarding the proposed mechanism of action, Liliopoulos et al. found that TNF-α increases the production of IL-6 and reduces the anti-inflammatory effect of the let-7 family by increasing the expression of *LIN28A* and *LIN28B*. Under physiological conditions, the let-7 family suppresses IL-6 expression directly, resulting in lower levels of IL-6 and alleviating NF-κB activation [19].

Conversely, both IL-1 and TNF-α have been shown to promote the production of IL-6 by myoblasts and myotube cells. Subsequently, IL-6 promotes continuous inflammation and the enhancement of NF-κB signaling [36,37,38,39]. IL-6 is a target for the let-7 family and predicted for nov-mmu-miRNA-1. Okamura et al. studied the effect of the overexpression and suppression of let-7e-5p on myosin heavy chain expression, glucose uptake, and mitochondrial activity in C2C12 myotube cells. They have proven that the downregulated expression of let-7e-5p inhibits *IGF2BP2* and results in atrophy in the C2C12 cell line [40]. Research presented by Sun et al. in mice models suggests that the let-7 family may be implicated in the old muscle phenotype, including atrophy and poor insulin signaling [41]. The hypothesized mechanism of nov-mmu-miRNA-1 was partially validated in our further experiments. We noticed that increasing the concentration of TNF-α significantly reduces let-7c,e,i expression, which was found common for inflammatory response and muscle atrophy by bioinformatics analysis. Noticeably, the expression of *LIN28B* gradually increased parallel with an increase in TNF-α correlation, and additionally, an inverse relationship between *LIN28B* and nov-mmu-miRNA-1 expression was observed. However, this phenomenon was not observed for *LIN28A*. Depending on treatment time, we did not find significant changes in let-7c,e,i and *LIN28A/B*. It is probably caused by the in vitro manner of our analysis, and the in vivo experiments could provide additional findings.

## 4. Materials and Methods

### 4.1. Cell Culture and Myotube Differentiation

The C2C12 murine myoblast cell line was obtained from ATCC (American Type Culture Collection, Manassas, VA, USA) and cultured in Dulbecco’s Modified Eagle Medium (DMEM) (PAN-Biotech, Aidenbach, Bavaria, Germany) supplemented with 10% fetal bovine serum (FBS) (PAN-Biotech, Aidenbach, Bavaria, Germany), 100 U/mL of penicillin, and 100 µg/mL of streptomycin. Culture was maintained at 37 °C in a humidified atmosphere with 95% air and 5% CO_2_. Cells were grown on uncoated plastic culture flasks to extract RNA. To stimulate myoblast differentiation into myotubes, DMEM with FBS was substituted for Differentiation Media (DM) containing 2% horse serum (PAN-Biotech, Aidenbach, Bavaria, Germany) and the previously indicated antibiotic combination for 5 days. Every 48 h, the DM was changed. Following differentiation, TNF-α was administered to the atrophy model cells, whereas free media was administered to the control cells. Every investigation set was performed in triplicates.

### 4.2. Atrophy Model

According to information obtained from the literature, C2C12 myotube atrophy was developed at various TNF-α concentrations and cell treatment periods. TNF-α concentrations between 10 and 100 ng/mL are often applied to generate atrophy models, with an entire treatment of 24 and 72 h. In our study, the myoblasts were cultured to 80–90% confluence before TNF-α treatment. In the discovery set, differentiated myotubes were stimulated once with 100 ng/mL of *mouse* TNF-α recombinant protein (Thermo Fisher Scientific, Waltham, MA, USA). This caused inflammatory-induced atrophy within 72 h. In the validation set, myotubes of the C2C12 cell line were exposed to the following TNF-α concentrations: 10, 50, 100, and 200 ng/mL for 72 h, and 100 ng/mL at 12, 24, 48, and 72 h time intervals. Then, o determine the expression of markers linked to muscle wasting (expression of *FBXO32* and *MuRF1*), cell viability tests, qRT-PCR, and microscopy evaluation were used to identify the atrophy [42].

### 4.3. miRNA-Seq and qRT-PCR Validation

Total RNA was purified from the harvested cells with a dedicated RNeasy Micro Kit (Qiagen, Toronto, ON, Canada) according to the manufacturer’s recommendations. The concentration and quality of obtained RNA samples were assessed, and a 100 ng/µL dilution of RNA was prepared. A total of 5 μL of a diluted RNA sample (100 ng) was used for the library preparation according to the manufacturer’s recommendations (QIAseq miRNA Library Kit, Qiagen, Hilden, Germany). Subsequently, RNA was ligated with 3′ and 5′ adapters, followed by revised transcription. After that, cDNA was size selected on a 6% polyacrylamide gel and was cleaned, quantified, and pooled. Finally, the cleaned miRNA library was eluted with 17 μL of nuclease-free water. Pooled libraries were loaded onto flow cells for cluster generation on-instrument. Next-generation sequencing (NGS) of miRNA was performed on an IlluminaMiSeq instrument (Illumina, Berlin, Germany). The reaction obtained fifty sequencing cycles. MiSeq Reporter software 2.5 for Fastq files generated was used. A total of 1 μL of obtained miRNA was reverse transcribed to cDNA in a 20 µL reaction according to the manufacturer’s protocol using a miRCURY LNA RT Kit (Qiagen, Hilden, Germany).

Quantitative PCR (qRT-PCR) was performed using miRCURY LNA miRNA PCR Assays (Qiagen, Hilden, Germany) combined with designed primers and miRCURY LNA miRNA Custom Probe PCR Assays (Qiagen, Hilden, Germany). Probes were designed on the request by the manufacturer for target sequences of nov-mmu-miRNA-1: 5′-cgagguagguugugugguu-3′ and nov-mmu-miRNA-2: 5′-acaaccauccucugcuacca-3′ (cat. no. 339351, Qiagen, Hilden, Germany). GAPDH was used as an internal control for PCR and data normalization (Qiagen, Hilden, Germany). A qPCR reaction was performed on a 96-well plate in 10 μL volume using a StepOne Plus device (Applied Biosystems, Foster City, CA, USA). Each well was filled with 2 µL of a diluted (1:40) RNase-free water cDNA template and an 8 μL reaction mix. The reaction conditions consisted of the following thermal cycles: 95 °C for 2 min, 40 cycles of 95 °C for 5 s, and 56 °C for 30 s. The level of miRNA expression was calculated using the 2^−ΔΔCt^ and 2^−ΔCt^ formulas. For each experiment, cell lines were cultured in triplicates. A one-way ANOVA test was used to compare differences in miRNAs’ expression levels between C2C12 cell cultures (treated and untreated). Results demonstrating *p*-values lower than 0.05 (*p* < 0.05) were considered statistically significant. Moreover, a false discovery rate (FDR) threshold of 0.05 was used to select miRNAs at different levels and for bioinformatics analysis purposes.

### 4.4. Bioinformatics Analysis

R software (v4.1.2; R Core Team 2021) and dedicated bioinformatics modules, as mentioned below, were applied. Using the miRDeep2 module, the new and known miRNA sequences were identified from miRNA-seq results, and their secondary and mature sequences were determined. Heat maps of the DEmiRNAs and DEnov-miRNAs expression log values and hierarchical clustering were conducted using Morpheus software version 6.1.0. A volcano plot was applied using VolcaNoseR version 2 software to display statistical significance (*p*-value) versus fold change to identify significant DEmiRNAs and DEnov-miRNAs in the discovery stage. Gene targets for nov-mmu-miRNA-1 were predicted by the miRDBase algorithm. WikiPathways analysis, Gene Ontology (GO), and Kyoto Encyclopedia of Genes and Genomes (KEGG) enrichment analysis were conducted using ShinyGO 0.80, GeneCodis, and Mouse Genome Informatics (MGI) tools. miRNet 2.0 software was applied to create an interaction network between genes targeted by nov-mmu-miRNA-1 and link them with inflammation and atrophy.

## 5. Conclusions

In conclusion, the experiments and bioinformatics analysis on the molecular relationship network partially explain the above hypothesis: a linkage between nov-mmu-miRNA-1, inflammation, and muscle atrophy. We are aware that our paper is not free from limitations. First, our experiments were performed using the myotube cell culture model without the involvement of other factors linked to the variability of cells involved in inflammatory response mediation. Second, the exact mechanism of mmu-miRNA-1 should be validated on the protein level with the application of a Western blot analysis. Further, in vivo experiments could be a definitive confirmation of our hypothesis. Nevertheless, a novel promising puzzle in miRNA machinery that controls inflammation and muscle wasting was identified. This fact encourages further analysis of this miRNA.

## Data Availability

The data presented in this study are available upon request from the corresponding author. The data are not publicly available due to privacy and ethical reasons.
